# An expert panel on the adequacy of safety data and physiological roles of dietary bovine osteopontin in infancy

**DOI:** 10.3389/fnut.2024.1404303

**Published:** 2024-06-11

**Authors:** Stephen A. Fleming, Sarah M. Reyes, Sharon M. Donovan, Olle Hernell, Rulan Jiang, Bo Lönnerdal, Josef Neu, Lawrence Steinman, Esben S. Sørensen, Christina E. West, Ronald Kleinman, John C. Wallingford

**Affiliations:** ^1^Traverse Science, Inc., Mundelein, IL, United States; ^2^Rev Bioscience, LLC, Boise, ID, United States; ^3^Department of Food Science and Human Nutrition, University of Illinois Urbana-Champaign, Urbana, IL, United States; ^4^Department of Clinical Sciences and Pediatrics, Umeå University, Umeå, Sweden; ^5^Department of Nutrition, University of California, Davis, Davis, CA, United States; ^6^Department of Pediatrics, Division of Neonatology, University of Florida, Gainesville, FL, United States; ^7^Departments of Pediatrics and of Neurology and Neurological Sciences, Interdepartmental Program in Immunology, Beckman Center for Molecular Medicine, Stanford University School of Medicine, Stanford, CA, United States; ^8^Department of Molecular Biology and Genetics, Aarhus University, Aarhus, Denmark; ^9^Harvard Medical School, Boston, MA, United States; ^10^Department of Pediatrics, Massachusetts General Hospital, Boston, MA, United States; ^11^Nutrispectives, LLC, Spokane, WA, United States

**Keywords:** infant, milk, osteopontin, safety, immunity, neurodevelopment, gastrointestinal

## Abstract

Human milk, due to its unique composition, is the optimal standard for infant nutrition. Osteopontin (OPN) is abundant in human milk but not bovine milk. The addition of bovine milk osteopontin (bmOPN) to formula may replicate OPN’s concentration and function in human milk. To address safety concerns, we convened an expert panel to assess the adequacy of safety data and physiological roles of dietary bmOPN in infancy. The exposure of breastfed infants to human milk OPN (hmOPN) has been well-characterized and decreases markedly over the first 6 months of lactation. Dietary bmOPN is resistant to gastric and intestinal digestion, absorbed and cleared from circulation within 8–24 h, and represents a small portion (<5%) of total plasma OPN. Label studies on hmOPN suggest that after 3 h, intact or digested OPN is absorbed into carcass (62%), small intestine (23%), stomach (5%), and small intestinal perfusate (4%), with <2% each found in the cecum, liver, brain, heart, and spleen. Although the results are heterogenous with respect to bmOPN’s physiologic impact, no adverse impacts have been reported across growth, gastrointestinal, immune, or brain-related outcomes. Recombinant bovine and human forms demonstrate similar absorption in plasma as bmOPN, as well as effects on cognition and immunity. The panel recommended prioritization of trials measuring a comprehensive set of clinically relevant outcomes on immunity and cognition to confirm the safety of bmOPN over that of further research on its absorption, distribution, metabolism, and excretion. This review offers expert consensus on the adequacy of data available to assess the safety of bmOPN for use in infant formula, aiding evidence-based decisions on the formulation of infant formula.

## Introduction

Optimal nutrition during the first year of life influences growth, susceptibility to infections, and the maturation of the gastrointestinal tract, immune system, and brain (1–4). Consistent evidence suggests that infants fed human milk (directly at the breast or by bottle) have more advantageous health outcomes than those fed formula (5–10). The benefits of human milk are attributed, in part, to its numerous biologically active components such as oligosaccharides, hormones, enzymes, cytokines, lactoferrin (LF), immunoglobulins, and milk fat globule membrane (1, 5, 11–16). It is believed the addition of such components to formula has potential to improve the health of formula-fed infants (17, 18). A joint workshop between the National Institutes of Health (NIH) and the Food and Drug Administration (FDA) was held to discuss an assessment framework surrounding the safe use of biologically active ingredients in infant formula (18). A post-meeting federal comment from the FDA noted that assessing the safety of such ingredients is complicated by the variability of human milk among mothers and across time, lack of consensus on follow-up periods in clinical trials, complexity of matrix effects, and lack of standardized approaches for evaluating the safety of bioactive ingredients (17).

Osteopontin (OPN) is one such protein that could be added to infant formulas to mimic the composition and functionality of human milk. OPN is particularly high in concentration in human milk compared to bovine milk (19), with growing evidence that its intake in early life supports immune, intestinal, and neural development (20–23). In 2022, the European Food Safety Authority (EFSA) published their scientific opinion that bovine milk osteopontin (bmOPN) is safe when added at a maximum concentration of 151 mg/L in infant formula (up to 6 months of age), follow-on formula (ages 6–12 months), and formula for young children (ages 1–3 years) (24). EFSA noted that while inconsistencies and limitations were present in the available science, they did not raise safety concerns (24). In the U.S., however, submission of the Generally Recognized as Safe (GRAS) Notice 716 for bmOPN was withdrawn after FDA concerns could not be alleviated in 2018 (25). The reasoning is stated in internal memos available at the CFSAN (Center for Food Safety and Applied Nutrition) FOIA (Freedom of Information Act) Electronic Reading Room on Bioactive Ingredients for Use in Infant Formula dated September 2020 (26). Since that submission and post-submission meetings thereafter (personal communication), the FDA noted concerns about bmOPN and its addition to infant formulas related to: high variability of dietary exposure to human milk OPN (hmOPN); relevance of the selected level of bmOPN in formula; lack of absorption, distribution, metabolism, and excretion (ADME) data; potential for crossing the blood–brain-barrier; its potential long-term immunomodulatory role and mechanism of action; its functional similarity to hmOPN; inadequacy of standard toxicological assessments for determining safety of bmOPN; and its potential relationship to various immune-related diseases.

Considering these concerns, a panel of experts convened to discuss the scientific evidence on the physiological roles and safety of orally ingested bovine milk OPN (bmOPN) for use in infant formulas for healthy term infants. The goal of the panel was to identify and interpret the research literature, identify what important gaps remain for determination of safe use of bmOPN in infant formula, and prioritize which gaps further research should address to inform future safety assessments. The efficacy of bmOPN was not of focus, however physiological effects that could potentially constitute or explain efficacy were in-scope. Here we report a narrative review on the physiological roles of milk OPN and evaluate existing data on bmOPN intake in early life, paying special attention to concerns raised previously by the FDA and at the FDA-NIH workshop.

## Panel procedures

Authors SAF, SMR, and JCW were not considered expert panel members. Experts were selected by authors BL and JCW for their direct research experience on OPN or their expertise regarding gastrointestinal and immune development, or neuroimmunology. All experts declared direct and related conflicts of interest to the subject material in the preceding 36 months, which are detailed in the author disclosures. All authors (except SMR) attended a two-day, in-person workshop to discuss the concerns listed (described above) by the FDA and to review the available evidence. The workshop was organized by Building Block Nutritionals and Arla Foods Ingredients. Those with published research on OPN (BL, RJ, ESS, CEW, SMD, LS) were invited to present their research, those without presented research on gastrointestinal/immune development (OH, JN), and all were encouraged to provide their interpretation of the data. RK was chosen to lead the panel and solicit alternative scientific opinions on the interpretation of the data. After the panel was concluded, SAF and SMR independently conducted a narrative review to fact-check the evidence presented, corresponding with experts to clarify discrepancies or solicit further discussion. Two post-workshop remote meetings were held to review and interpret the evidence. A draft manuscript was written by SAF and SMR and reviewed over 3 rounds by all authors. As a final check against bias, SAF solicited alternative opinions on the interpretation of the data and conclusions of the panel on a 1:1 basis, such that responses remained anonymous to all authors except SAF. Experts were asked to provide their final opinion on the sufficiency of the data as it relates to safety, their interpretation of specific elements of the research, what gaps remain, and what future research to prioritize. Experts were not asked to provide a final conclusion on whether or not bmOPN is safe for use in infant formula. All authors were compensated by Arla Foods Ingredients for their efforts to attend the workshop and draft, revise, or edit the manuscript. Neither Arla Foods Ingredients nor Building Block Nutritionals were permitted to participate in the remote post-meeting workshops, nor to hold contributor or editorial roles in the writing of the manuscript.

## Narrative review

The topics reviewed by the panel included dietary exposure to milk OPN, ADME of consumed milk OPN, OPN’s role in gastrointestinal, immune, and neural development, the extent of the functional bioequivalence between forms of OPN (whether dietary intake of human, bovine, or recombinant forms of OPN demonstrate similar ADME profiles and physiological effects), and potential matrix effects with other components in infant formula and human milk. Although not a systematic review, study eligibility criteria (Table 1) were developed to guide the panel’s assessment of data toward that of the appropriate developmental stage (infancy), type of exposure to OPN (dietary), health status (no pre-existing diseases, disorders, or injuries, excepting prematurity), and study design (*in vivo* research).

**Table 1:** Study eligibility criteria.^1^

Inclusion criteria	Exclusion criteria
Population:^1^ *In vivo* trials in humans conducted in healthy pediatric populations or animal models of healthy early-life development.	Population: *In vivo* trials in adult humans or animals models of adulthood. Populations/models diagnosed with, at-risk for, or as a model of, any pre-existing disease, disorder, or injury, except for prematurity. *In vitro* trials in any model that examine the function of bmOPN, excepting those used to examine potential matrix effects.
Exposure:^1^ Intake of any form of OPN (human, bovine, murine, recombinant, or otherwise) in any matrix (e.g., water, formula, milk), delivered using any dietary method (e.g., *ad libitum* feeding, oral gavage, or enteral feeding) that does not bypass the gastrointestinal tract.	Exposure: Studies in which delivery of OPN bypassed the gastrointestinal tract, such as through i.p. injection, parenteral nutrition, intranasal delivery, or other methods.
Outcomes: Outcomes related to exposure, ADME, anthropometric growth, immune function, brain development, cognition, or matrix effects with other milk components.	Outcomes: Studies assessing the function of endogenous OPN as an outcome in response to an intervention (e.g., OPN expression in response to injury or inflammation). Correlations between endogenous OPN and other physiological effects.
Study Design: *In vivo* research	Study Design: *in vitro* or *ex vivo* trials

^1^Exceptions were made for any criteria in order to describe endogenous concentrations of OPN in various fluids (e.g., milk, plasma) across the lifespan. ADME, absorption, distribution, metabolism, excretion; bmOPN, bovine milk osteopontin; OPN, osteopontin.

Where appropriate, data that fit exclusion criteria were still used to describe exposure (e.g., endogenous levels of plasma OPN across the lifespan), and concepts related to its structure. It is well established that mechanisms of gastrointestinal, immune, and brain development are distinct from those supporting mature function in adulthood (27–30), thus the panel concluded that data generated in adult animals were not translatable to understanding the safety of bmOPN consumed by infants and were thus not reviewed. Then, the panel concluded that the inclusion of data generated on OPN in non-dietary contexts (including *in vitro* research) or in models of disease would not inform its function in a healthy, developmental, dietary context. For example, literature describing endogenously produced OPN or non-dietary OPN (e.g., administered by i.p. or i.v. injection) in adult models of inflammatory disease (31, 32) were not reviewed. Lastly, studies in preterm pig models were also included as they are relevant to exposure to OPN in early development.

Throughout, language denoting a “difference” or “change” between groups are only those that were statistically significant (as defined by the original authors, typically *p* < 0.05), unless otherwise stated. Likewise, the lack of a difference indicates a comparison made where no statistical significance was reached, unless otherwise stated.

## Infant exposure to dietary osteopontin from milk and formula

**Figure 1:**
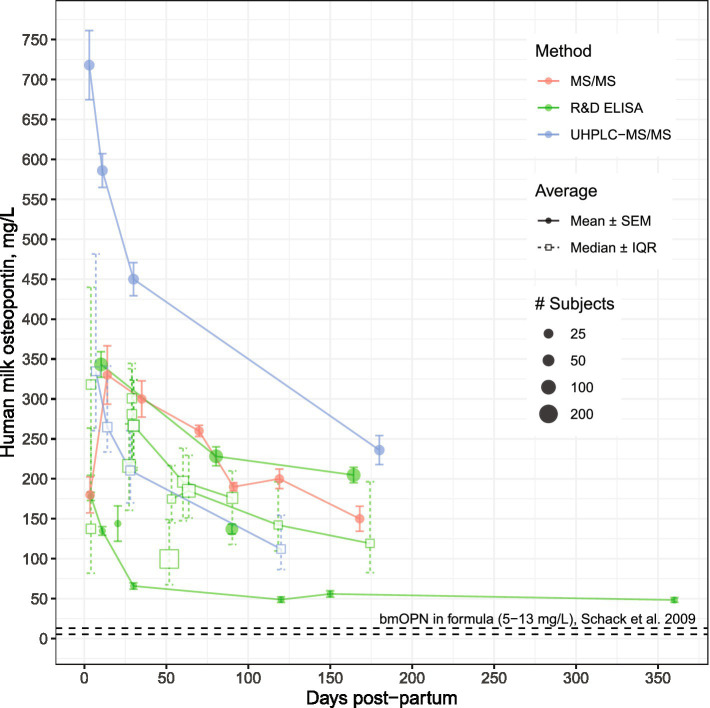
Osteopontin concentrations in human milk from mothers of healthy, term infants by analytical method. Modified from Sørensen and Christensen (44). Data used for visualization can be found at https://github.com/Traverse-Science/Osteopontin-Expert-Panel. For studies reporting samples collected across a range (e.g., 0–3 months post-partum), the midpoint was used. Concentrations of hmOPN are high at birth and gradually decline over the first 6 months of lactation. Points connected by a line represent longitudinal assessments from the same study. bmOPN, bovine milk osteopontin; IQR, Interquartile range; MS, mass spectrometry; R&D ELISA, enzyme-linked immunosorbent assay from R&D Systems (Abingdon, UK); SEM, Standard error of the mean; UHPLC, Ultra-High-Performance-Liquid Chromatography.

OPN, found ubiquitously in nearly all body fluids, is specifically expressed at high levels from mammary tissue during lactation (33). Although hmOPN was originally estimated to be almost 10% of total protein in human milk (34), Schack et al. later reported term human milk to contain 138 mg/L hmOPN, about 2.1% (wt/wt) of the total protein in mature milk (19). Since 2009, several other studies have reported concentrations of hmOPN (23, 35–40). Collectively, these new data confirmed the observation that hmOPN concentrations decrease over the course of lactation, a trajectory also observed in murine and bovine milk (41, 42), suggesting a conserved biological pattern across mammals. The concentration of hmOPN is correlated with that of total protein, α-lactalbumin, LF, and casein (43). hmOPN is present at 250–350 mg/L in colostrum, declining to around 65–250 mg/L in mature, term human milk (Figure 1). For comparison, standard infant formulas (predominantly bovine milk-based) contain considerably less OPN (~9–15 mg/L of bmOPN) than is present in human milk (19).

Recent studies provide evidence that hmOPN concentration varies with many maternal factors including diet, parity, age, ethnicity and body composition (36, 40, 43). For example, higher levels of hmOPN were observed in women who delivered vaginally, had a non-obese postpartum BMI (<30 kg/m^2^), and abstained from smoking (36). Additionally, among these women, low to moderate inverse associations were found between hmOPN and maternal intake of daily energy, and grams of fat, carbohydrate, and fiber intake (36). While variation in hmOPN appears high, most variation comes from between-study sources, with less variance within studies. Variation is even less pronounced when considering studies that use the same measurement method, such as ELISA versus HPLC (Figure 1). Notably, the human OPN (hOPN) ELISA from Immuno-Biological Laboratories (Gunma, Japan) has been reported to overestimate OPN concentrations compared to the hOPN ELISA from R&D Systems (Abingdon, UK) (19, 44). Given the heterogeneity in study design and assay method, it was not possible to ascertain true geographic differences in hmOPN concentration between mothers from Japan (45, 46), Denmark (19), the U.S. (23, 47, 48), Turkey (36), and China (38, 39, 43).

**Panel conclusions** The panel concluded that the existing data enable quantification of hmOPN across the first six months of life.

## Absorption, distribution, metabolism, and excretion of milk osteopontin

The structure and digestion of OPN from human and bovine milk have been extensively reviewed (21, 44). Briefly, OPN is a highly acidic protein that lacks a fixed tertiary structure (i.e., is intrinsically disordered) and undergoes extensive post-translational modifications including phosphorylation and glycosylation (49). There are 3 main splice variants of the protein: OPNa (the full-length form), OPNb (lack of exon 5), and OPNc (lack of exon 4), with OPNa being the only variant expressed in human milk (50). hmOPN and bmOPN are comprised of 298 and 262 amino acid residues, respectively, and are highly homologous with identical amino acids on 182 positions in addition to 44 structurally conserved amino acid substitutions (44). Full-length OPN undergoes cleavage by endogenous proteases in milk, resulting in several N-terminal-derived fragments (51, 52). C-terminal fragments are not detected in human and bovine milk, as they are likely further degraded to smaller peptides by proteases in milk (51, 52).

*In vitro* and *in vivo* studies have demonstrated that oral OPN is resistant to gastric and intestinal digestion. For example, human and bovine OPN have been detected intact after incubation with newborn gastric aspirates for 1 h at pH 3 (53). Additionally, intact bmOPN was found in both stomach and small intestinal contents of OPN knock-out (KO) mouse pups 30 min after oral gavage (54–56). Resistance to digestive proteases is primarily attributed to the glycosylated and conserved threonine residues close to the Arg-Gly-Asp integrin-binding sequence (44, 54, 57, 58). *In vitro* evidence suggests that intact OPN and/or fragments can cross the intestinal barrier via transcytosis (59), and dietary bmOPN has been detected in the plasma of rodents and humans (23, 55, 60), demonstrating absorption.

**Figure 2:**
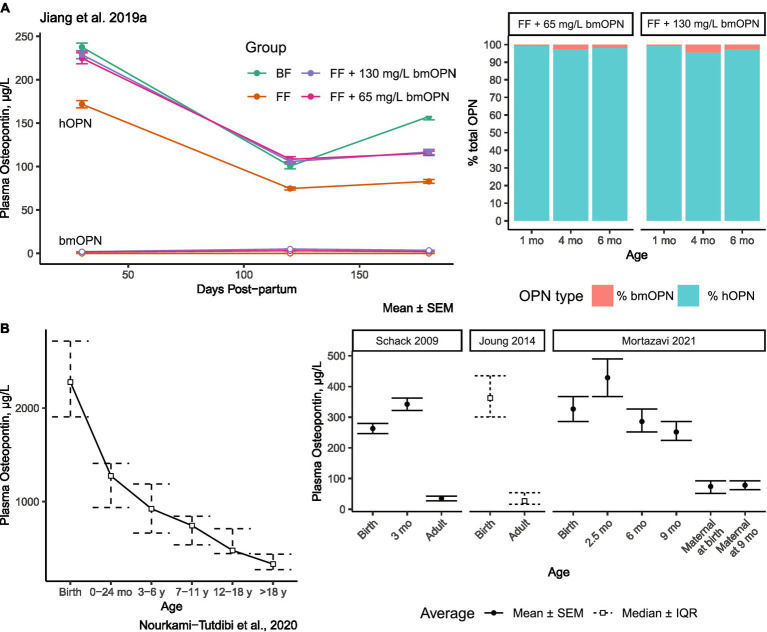
Concentrations of OPN in plasma in human infants and across the lifespan. **(A)** Concentration of bmOPN and hOPN among infants breastfed or fed formula supplemented with bmOPN Open points represent bmOPN, solid points represent hOPN. Stacked bars indicated the proportion of total OPN from bmOPN or hOPN. bmOPN represented less than 5% of total OPN at the greatest. **(A)** Modified from Jiang et al. (23). **(B)** Concentrations of plasma hOPN across the lifespan Modified from Nourkami-Tutdibi et al. (67); Schack et al. (19); Joung et al. (65); Mortazavi et al. (66). BF, breastfed; bmOPN, bovine milk osteopontin; FF, formula-fed; hOPN, human osteopontin; mo, Month.

Assessing the distribution of dietary bmOPN is complicated by its ubiquitous endogenous presence in numerous tissues and fluids (21, 22, 31, 33, 44, 50, 61, 62). While human milk is particularly rich in OPN, plasma is not. The concentration of OPN in mature human milk ranges between 65–250 mg/L (Figure 1), term infant urine contains 6.6 mg/L (27 mg/L in adult urine) (63), adult cerebrospinal fluid contains 0.319 mg/L (64), term infant plasma contains between 0.075–0.170 mg/L (23), and adult plasma the lowest of these estimated at ≤0.080 mg/L (19, 65, 66). In infancy, the circulating concentration is a combination of dietary OPN and endogenously synthesized OPN. Jiang et al. reported ~75–170 μg/L hOPN in plasma of formula-fed infants compared to ~100–238 μg/L hOPN in plasma of breastfed (BF) infants (23) (Figure 2A). The presence of hOPN in plasma of formula-fed infants who consumed no hmOPN (at ~75% of the plasma concentration of hOPN in breastfed infants), suggests that the majority of circulating hOPN is endogenous in origin. Endogenous hOPN may be increased in response to dietary intake of bmOPN, as plasma concentrations of hOPN were higher in infants fed formula containing 65 or 130 mg/L bmOPN than those fed formula without supplementation of bmOPN (Figure 2A). This study also reported that bmOPN was detected in circulation but represented 1.75–5% of total plasma OPN, even among infants receiving 130 mg/L bmOPN-enriched formula (Figure 2A).

Results from multiple studies demonstrate that the concentration of plasma hOPN is high at birth and gradually declines over the lifespan (Figure 2B) (67). By adulthood, plasma hOPN concentrations fall to between 26–80 μg/L (19, 65) (Figure 2B). Although absolute concentrations differ (representing the use of different ELISAs), the trend of decreased OPN from birth to adulthood is replicated (19, 65, 67). For comparison, circulating hOPN is about 1,000x lower in concentration than that found in human milk.

**Figure 3:**
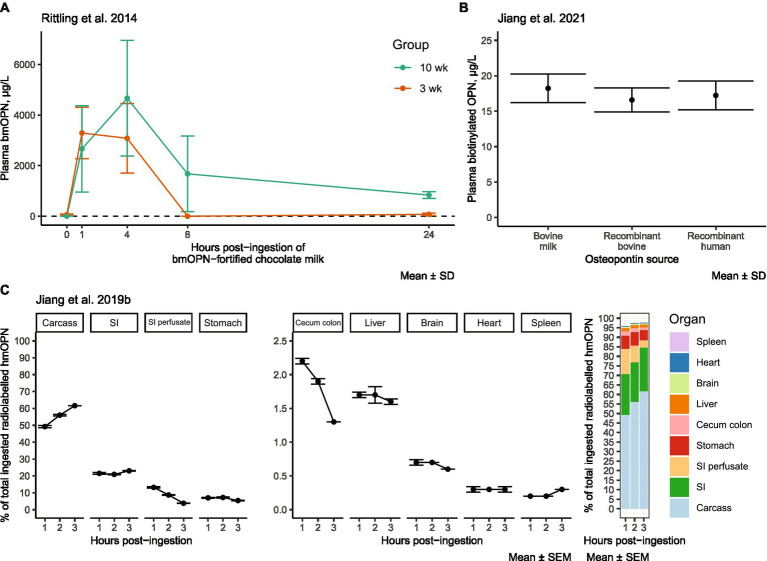
Experimental evidence of the distribution of dietary OPN: **(A)** bmOPN in plasma of OPN-deficient KO mice after oral gavage of 50 mg bmOPN in chocolate milk. Modified from Rittling et al. (60). **(B)** Plasma levels of biotinylated OPN 3 h after feeding (oral gavage) PND 12 WT mice with 12 mg OPN/kg bodyweight/day from bovine milk (Lacprodan OPN-10, Arla Foods Ingredients, Viby, Denmark), algal recombinant bovine OPN (Triton Algae Innovations, San Diego, CA), or algal recombinant human OPN (Triton Algae Innovations, San Diego, CA) in water. Modified from Jiang et al. (55). **(C)** Tissue distribution of [125I] radio-labeled hmOPN 1–3 h after oral gavage in WT mice. Modified from Jiang et al. (41). bmOPN, bovine milk osteopontin; hmOPN, human milk osteopontin; KO, knockout; PND, postnatal day; SD, standard deviation; SEM, standard error of the mean; SI, small intestine; wk., week; WT, wild-type.

Insights into the distribution and metabolism of dietary OPN have emerged from studies conducted in mice. In an acute absorption study conducted by Rittling et al., OPN-deficient mice were fed milk enriched with bmOPN at 250 mg/mL, for a total one-time dose of 50 mg. Peak plasma levels of bmOPN (measured by an in-house competition ELISA; Rittling et al. suggest these were likely peptides) were observed at 1 and 4 h in 3-and 10-week-old mice, respectively (60) (Figure 3A). The levels of plasma bmOPN rapidly declined to an undetectable level between 4 and 8 h in 3-week-old mice, with low levels (compared to peak concentrations) detectable in 10-week-old mice. bmOPN appears to be rapidly (within 8 h) cleared from plasma, though whether that be in response to tissue uptake or metabolism is unclear. There were similar levels of biotinylated forms of bmOPN, recombinant human OPN, and recombinant bovine OPN in plasma 3 h after feeding in PND 12 mouse pups (55) (Figure 3B), which may represent peak levels (60).

After 1 to 3 h following oral gavage with radio-labeled hmOPN, label was detected in multiple organs and tissues of the mouse (41) (Figure 3C). After 3 h, >95% of the label was recovered, with ~62% found in the carcass, ~23% in the small intestine, 5.4% in the stomach, and ~ 3.8% in intestinal contents. The remaining ~6% was present at amounts of <2% in the cecum and liver, and < 0.6% in the brain, spleen, and heart. Percentages reflect total label measured, unadjusted for tissue size. Over the course of 3 h, the labeled signal increased in carcass tissue, decreased in small intestinal perfusate and cecum colon, and remained relatively stable in other tissues (41). Further research is needed to characterize whether orally ingested OPN has a distinct metabolic fate from that of endogenous OPN. It remains an open question if, to what extent, and how dietary OPN crosses the blood–brain barrier (BBB). Given that the hmOPN was radiolabeled with iodine-125 (41), which incorporates into tyrosine and histidine residues, it is not clear if the radiolabeled signal in these tissues is intact/partial hmOPN, smaller peptides and free amino acids thereof, or the same residues synthesized into new proteins. Osteopontin peptides have been detected in the blood of human subjects as early as 30 min or up to 7 h after consumption (68), suggesting the bmOPN signal detected by Jiang et al. within 3 h of bmOPN ingestion (41) could have represented absorption of OPN-related peptides in blood. It is also possible that the signal measured was indeed intact hmOPN, but the protein was located within blood or neurovascular tissue without crossing the BBB, as whole brain samples were not perfused prior to measurement, and thus contained blood. Regardless, whole brain lysates from OPN KO pups (devoid of tissue OPN) fed milk from wild-type (WT) dams demonstrate the presence of OPN (measured by Western blot) (41). The panel concluded that there is some evidence from the KO model that BBB transport of dietary OPN can occur, which may apply to endogenously produced OPN from serum as well. More research is required to definitively conclude that transport does occur and the physiological importance of such transport.

## ADME summary

- In humans, OPN is 382-3333x higher in mature human milk, 39-88x higher in term infant urine, and 2-4x higher in adult cerebrospinal fluid as compared to term infant plasma (23, 63, 64). Plasma OPN is greatest in infancy and reduces across the lifespan to its lowest levels in adulthood (19, 65–67).
- bmOPN is resistant to gastric and small intestinal digestion, in mice (53–56).
- After acute intake of labeled hmOPN in mice, most of the label is found in carcass tissue (62%), small intestine (23%), stomach (5%), small intestinal perfusate (4%), with <2% each found in the cecum, liver, brain, heart, and spleen (41).
- It is unclear if, how, and to what extent OPN crosses the BBB.
- In 3-week-old mice, acute intake of bmOPN largely clears circulation within 8–24 h (60).
- After intake of bmOPN or algal recombinant human/bovine OPN, the forms of each are present in similar levels in the plasma of mice (55).
- In infants, bmOPN is present in circulation at 1.75–5% that of endogenous human OPN with daily consumption. Consumption of bmOPN may stimulate an increase in endogenous circulating OPN (23).
- Gaps in the ADME profile include clarification of transport across the BBB, quantification of dietary bmOPN’s distribution in tissues and fluids relative to the concentration of endogenous OPN, quantification of its half-life, and replication of the effects shown thus far.

**Panel conclusions** Although gaps in the understanding of the ADME profile of bmOPN exist, the panel questioned the utility of ADME as a paradigm to contextualize the physiological function or developmental impact of dietary bmOPN, given its low concentration in plasma relative to endogenous OPN. The panel suggested further research on clinically relevant outcomes be prioritized above ADME.

## Orally ingested milk osteopontin and development

The FDA notes a need to standardize approaches for evaluating the safety of bioactive ingredients for use in infant formula (17). The FDA’s state that, especially for potentially immunomodulatory substances, standard toxicological endpoints may not inform whether bmOPN is safe for use in infant formula or not (26). In an attempt to address this need, a previous expert panel separated safety endpoints related to immunity into those that are clinically relevant standard endpoints and those that are candidate markers of immune development (69). Clinically relevant endpoints included (but were not limited to) outcomes such as anthropometrics, incidence of infection, adverse events related to inflammation, vaccine response, hospitalizations, fevers, atopic dermatitis, and food allergy. Biomarkers included measurements of cytokines and immune cell populations. Callahan et al. recommended that the standard clinical endpoints are sufficient to demonstrate safety of novel bioactive ingredients for immune-related outcomes, and that the biomarkers can be used as indicators of stereotypical immune development (69). Using a range of clinically relevant outcomes and physiological biomarkers, 10 preclinical studies using either genetic knock outs or oral supplementation have investigated the role of dietary OPN and potentials mechanisms of action across gastrointestinal, immune, and neurodevelopmental outcomes. These include four KO studies in mice (41, 54–56); 1 rat trial (70); 1 term pig trial (71); 3 preterm pig trials (72–74); and 1 study in rhesus monkeys (75). Of those that measured bodyweight (BW) or anthropometrics, none reported an effect of OPN supplementation on growth (41, 55, 70–72, 75).

### The role of dietary OPN examined in OPN knock-out mouse models

Jiang et al. report several cross-fostering experiments using WT and OPN KO mice to assess gastrointestinal development (54–56), neurodevelopment (41, 54, 55), and immunity (54, 55). In these experiments, WT or KO mouse pups (Pup^WT^, Pup^KO^) were cross-fostered to nurse WT or KO dams (Dam^WT^, Dam^KO^), creating up to 4 groups: Pup^WT^/Dam^WT^ (a model replicating breastfed infants), Pup^WT^/Dam^KO^ (a model replicating formula-fed infants), Pup^KO^/Dam^WT^, and Pup^KO^/Dam^KO^ groups, respectively. The Pup^WT^/Dam^WT^ group represents exposure to both endogenous and dietary OPN, and the Pup^WT^/Dam^KO^ group represents exposure only to endogenous OPN. While not direct evidence of the safety of bmOPN, such models provide insight into the function of dietary OPN in early-life. Pups nursed to WT dams were exposed to an average of 12 mg OPN/kg BW/day (55) from milk until weaning on postnatal day (PND) 21 (41, 54–56). Milk yield was similar between Dam^WT^ and Dam^KO^ (41) mothers. Pup^WT^/Dam^KO^ pups had lower jejunal cell proliferation (56), smaller inner surfaces of the jejunum on PND 10 and 20 (54–56), but not post-weaning on PND 30 (56) compared to Pup^WT^/Dam^WT^ pups. Absolute length of the intestines was unchanged on PND 10 in 2 studies (54, 55), and shorter in Pup^WT^/Dam^KO^ pups on a per BW basis from PND 4–6 and equivalent from PND 8–30 (56). Post-weaning at PND 30 Pup^WT^/Dam^KO^ pups had lower alkaline phosphatase activity in the brush border of the duodenum and jejunum as well as fewer goblet cells, enteroendocrine cells, and Paneth cells compared to Pup^WT^/Dam^WT^ pups (56). mRNA expression of the integrins α_V_, β_3_, and CD44 were generally lower in Pup^WT^/Dam^KO^ compared to Pup^WT^/Dam^WT^ between PND 4–20, but equivalent on PND 30. In contrast, protein expression of the same proteins was lower in Pup^WT^/Dam^KO^ compared to Pup^WT^/Dam^WT^ pups on PND 10, 20, and 30 (56). Moreover, compared to Pup^WT^/Dam^WT^ pups, Pup^WT^/Dam^KO^ pups had lower expression between PND 10 and PND 30 of several proteins related to signaling pathways important for intestinal development, namely extracellular signal-regulated kinase (ERK), phosphoinositide-3-kinase/protein kinase B (PI3K/Akt), Wnt, and focal adhesion kinase signaling (56, 76–78). Together, these data provided evidence that the absence of OPN in milk can alter some mechanisms of intestinal development, some of which remained altered after weaning and cessation of OPN intake.

Jiang et al. demonstrated that the absence of orally ingested milk OPN can lead to suboptimal myelination patterns and impaired performance on behavioral tasks measuring motor learning and memory (41). Although mRNA expression of OPN in whole-brain lysates of pups did not differ significantly between groups, compared to Pup^WT^/Dam^WT^ pups, Pup^WT^/Dam^KO^ pups had significantly lower concentrations of OPN protein in whole brain lysates on PND 6 and 8, a period considered critical for brain development (41). Immunohistochemistry revealed that Pup^WT^/Dam^KO^ pups also had reduced proliferation and differentiation of glial cells into oligodendrocytes, fewer OPN+ cells, and fewer myelin basic protein (MBP) and myelin-associated glycoprotein (MAG) positive cells detected in the hippocampus, corpus callosum, striatum, and cerebellum as compared to Pup^WT^/Dam^WT^ pups between PND 6–20. These were concurrent with thinner myelin sheaths in the spinal cord on PND 8 in Pup^WT^/Dam^KO^ pups compared to Pup^WT^/Dam^WT^ (41). Of the outcomes measured after weaning, there were no differences in mRNA expression of MBP, MAG, proteolipid protein, 2′,3′- cyclic nucleotide 3′-phosphodiesterase, or myelin oligodendrocyte glycoprotein, or protein expression of MBP, MAG, or ERK-1/2 and PI3K/Akt signaling pathways. Protein expression of neural/glial antigen 2 (a glial cell marker) and anti-adenomatous polyposis coli clone 1 (an oligodendrocyte marker) remained reduced in Pup^WT^/Dam^KO^ pups compared to Pup^WT^/Dam^WT^ after weaning, as well as behavioral performance on the passive avoidance and rotarod tasks, measures of memory and motor learning. Thus, lack of exposure to early-life OPN had transient impacts on markers of myelination and expression of OPN, and post-weaning impacts on markers of glial cell development and behavior.

Regarding immunity, two studies demonstrated that Pup^WT^/Dam^KO^ weaned on PND 21 have increased plasma TNF-α in response to intraperitoneal injection of lipopolysaccharide (LPS) from *Escherichia coli* on PND 30 as compared to Pup^WT^/Dam^WT^ (54, 55), indicating an increased response to immune challenges.

In summary, KO models confirmed that pups consuming dam’s milk without OPN had altered gastrointestinal and neural physiology, impaired cognitive performance, and increased immune responses when compared to pups consuming dam’s milk with OPN. Some of these changes remained after weaning and cessation of OPN intake. Such studies provided evidence that intake of milk OPN may play a role in development. Further research is needed to assess whether such effects extend to bmOPN supplementation, and not its absence.

### Supplementation with bovine osteopontin

Throughout, references to the concentration of bmOPN refer to the concentration of the protein, and not the ingredient/source used. All studies used Lacprodan OPN-10 (Arla Foods Ingredients, Viby, Denmark) as the source of bmOPN, which was reported by the original publications or confirmed through personal communication with the manufacturer (70). Lacprodan OPN-10 is composed of ~80% protein, 9% ash, 4% moisture, and ~ 0.1% lactose. bmOPN represents over 88.5% of protein, with 25.4–26.5% of bmOPN in the product as full-length OPN, with 73.5–74.6% an N-terminal fragment, and no C-terminal fragments. A more detailed description of the composition and specifications is available elsewhere (24).

Using the same KO models and study design, Jiang et al. orally gavaged Pup^WT^/Dam^KO^ pups with 12 mg bmOPN/kg BW/day in water (54). Supplementation of Pup^WT^/Dam^KO^ with bmOPN compared to Pup^WT^/Dam^KO^ without bmOPN increased whole-brain MBP and MAG, increased the villus height to crypt depth ratio, and post-weaning attenuated impairments in cognitive performance and lowered the level of plasma tumor necrosis factor α (TNF-α) in response to LPS, bringing the overall phenotype of bmOPN supplemented pups closer to that of Pup^WT^/Dam^WT^ pups (54, 55). The same effects were found with supplemental algal recombinant bovine OPN (Triton Algae Innovations, San Diego, CA), or algal recombinant human OPN (Triton Algae Innovations, San Diego, CA), demonstrating functional similarity on these outcomes between forms of OPN despite their different post-translational modifications (the recombinant forms were not glycosylated and contained fewer phosphorylation sites than bmOPN) (55).

Chen et al. studied the effects of bmOPN supplementation on the adaptive immunity of Sprague Dawley rats by gavaging them with a standard (Biostime Beta-star, Biostime [Guangzhou] Health Product Company Ltd., China, containing 10 mg/L bmOPN) or bmOPN-enriched formula (Biostime Pi-star, Biostime [Guangzhou] Health Product Company Ltd., China, containing 65 mg/L bmOPN from Lacprodan OPN-10) in addition to nursing and compared them to an exclusively dam-fed group for 21 days (PND 7–28) (70). On PND 28 those exclusively dam-fed had higher concentrations of CD8+ T cells in lymph nodes compared to those fed standard formula, with no significant differences between bmOPN-enriched and dam-fed groups. Rats fed bmOPN-enriched formula had greater CD3+ cells in lymph nodes, but not in the spleen as compared to rats fed a standard formula. No significant differences were observed in CD4+, CD8+, or B220+ cell concentrations in lymph nodes or spleen between formula groups. After weaning, the immunoglobulin (IgG, IgA, and IgM) response to LPS was not different between formula groups, however anti-ovalbumin IgG (but not IgA or IgM) was increased in the bmOPN fed rats, shifting their response toward that of the dam-fed rats. Chen et al. interpreted these data as evidence that bmOPN-enriched formula promotes differentiation of CD3^+^ T cells and the T-cell-dependent humoral immune response, with 65 mg/L bmOPN resulting in modest changes in immune markers and shifting the phenotype closer to that of nursed pups (70).

While the data on bmOPN supplementation in both mouse KO and WT models suggest numerous physiological functions of dietary bmOPN, large animal data have not reproduced such effects. Artificially reared term pigs were fed a soy-protein-isolate-based milk replacer (to eliminate residual bmOPN in bovine-milk-based replacers) supplemented with Lacprodan OPN-10 at 250 mg OPN/L (estimated 25–71.1 mg bmOPN/kg BW per day) in milk replacer from PND 3–32 (71). Magnetic resonance imaging revealed some differences in regional brain volumes, microstructure of the corpus callosum, and minor differences in behavior. While the authors initially hypothesized OPN supplementation would improve neurodevelopment, they concluded that the results were minimal and did not report any impairments to neurodevelopment (71).

Three studies used the preterm pig model, in which pigs were delivered via cesarean section between 89–92% of their full gestational length (115–117 days) (72–74). Pigs delivered preterm were reared individually in heated incubators and provided supplemental oxygen (72–74). They were fed via parenteral nutrition and gradually (72, 74) or entirely (73) transitioned to enteral nutrition. Given the lack of exposure to colostrum, pigs were provided maternal plasma to support passive immunity (72, 73).

Aasmul-Olsen et al. report that preterm pigs fed raw bovine milk supplemented with bmOPN (Lacprodan OPN-10, 46 mg bmOPN/kg BW per day, median 319 mg/L from PND 1–19) had higher villus height-to-crypt ratios on PND 19 compared to pigs fed raw bovine milk without supplementation (72). However, OPN-supplementation did not result in significant differences in the number of proliferative enterocytes on PND 19. For markers of immunity, OPN supplementation had low to modest effects. OPN-supplemented pigs had higher blood concentrations of monocytes and lymphocytes on PND 8, and a lower neutrophil phagocytic rate at PND 19 compared to pigs fed raw bovine milk without supplementation (72). However, there were no differences in T cell subsets including T-helper cells, cytotoxic T cells and regulatory T cells, and concentrations of interleukin-10 (IL-10) and TNF-α cytokines were similar in LPS-stimulated whole blood samples and undetectable in unstimulated samples (72). Among brain-related outcomes, there were no differences in whole and sub-region brain weights at euthanasia on PND 19, nor differences on open-field behavior, early motor development, or spatial learning on PND 12–18 compared to controls (72).

In another study, preterm pigs were exposed to prenatal inflammation via intra-amniotic LPS injection 3 days prior to birth and fed bmOPN (Lacprodan OPN-10, 2.22 g/L, 53.3 mg bmOPN/kg BW per day) for 5 days (74). Those fed bmOPN-enriched formula had no differences in clinically relevant endpoints of inflammatory intestinal injury or incidence of diarrhea compared to the control formula. Other biomarkers also did not differ, including: intestinal villus/crypt height/depth, lactase activity, distal/colon goblet cell density, colonic microbiota composition, blood chemistry, serum glucose, galactose, or iron, helper T cell concentrations, IL-1ß, lymphocytes, monocytes, neutrophils, or neutrophil phagocytic capacity, or time-to-stand (an indicator of motor development at PND 5) (74).

Additionally, Møller et al. enterally supplemented (in addition to parenteral nutrition) preterm pigs with 2 g bmOPN/L (Lacprodan OPN-10) in water at a rate of 5 mL/kg BW (10 mg bmOPN/kg BW) every 3 h from birth through PND 2, totaling 80 mg bmOPN/kg BW per day (73). Upon switching to enteral nutrition, preterm pigs were fed every 3 h with 15 mL formula/kg BW, with bmOPN supplemented pigs receiving 2.22 g bmOPN/L formula (266.4 mg bmOPN/kg BW per day) for 1.5 days. The high dose of bmOPN was used to replicate the concentration of OPN in colostrum, rather than mature milk. The authors reported reduced severity of inflammatory intestinal injury and greater absorption of mannitol in premature pigs fed bmOPN-enriched formula compared to those fed a standard formula (73). Otherwise compared to controls, bmOPN did not affect villus height; enzyme activity of lactase, maltase, sucrase, aminopeptidase, aminopeptidase N, or dipeptidyl-peptidase IV; or galactose absorption.

Donovan et al. found that the jejunal transcriptome of intestines from BF rhesus monkeys was distinct from those fed a commercially available infant formula (75). Monkeys fed the same formula supplemented with 125 mg/L bmOPN (Lacprodan OPN-10, intake on a BW basis not available) from birth to 3 months displayed an intermediate phenotype between BF monkeys and those fed un-supplemented formula in a primary cluster containing 50% of the genes. That module included genes related to functions including but not limited to: cell adhesion, cytoskeleton remodeling, neuronal development, protein modification, and the cell cycle (75). No differences in red blood cell concentrations, hemoglobin, hematocrit, white blood cells, or differential white blood cell counts were observed.

### Summary of preclinical evidence

Of the four KO mouse studies (41, 54–56), one rat study (70), one term pig study (71), three preterm pig studies (72–74), and one monkey study (75), none have reported any effect of bmOPN supplementation on body growth or anthropometrics, nor any adverse effects related to supplementation. Genetic KO studies in mice demonstrate that WT pups consuming milk with no OPN have impaired cognitive performance, increased immune responses to LPS, and altered gastrointestinal and neural physiology compared to WT pups consuming milk with OPN (41, 54–56). Supplementation with bmOPN, recombinant human, or recombinant bovine OPN can prevent/attenuate many of these effects, demonstrating functional similarity between forms (54, 55). Rats fed bmOPN-enriched formula in early life did not have an altered immunoglobulin response to LPS after weaning, but their response to ovalbumin shifted from those fed a standard formula toward that of dam-fed rats. bmOPN-feeding resulted in modest changes in T cell related immune markers (70).

bmOPN supplementation in a soy-based formula to artificially-reared term pigs had minimal impacts on neurodevelopment (71). Studies in preterm pig models were heterogenous with respect to the dose and duration of exposure, with exposures to bmOPN 2-20x higher than OPN in mature human milk (72–74). No study reported an impact of bmOPN supplementation on brain development. bmOPN had a modest effect, if any, on markers of gastrointestinal and immune development. bmOPN supplementation in bovine-milk-based formulas to Rhesus monkeys altered the jejunal transcriptome with no impacts on hematology-related parameters (75).

**Panel conclusions**

- The heterogeneity in study designs and lack of consistent physiologic effects within and across species calls into question under what contexts dietary bmOPN has an immunomodulatory role for which a mode of action could be established.
- Some panel members concluded that the effects were modest and do not require further investigation.
- Some panelists suggested that further animal research should strive to reproduce the effects shown using longer study designs that replicate the first 12 months of human infant life. To further characterize potential immunomodulatory effects of bmOPN, using the response to vaccines or viral/bacterial pathogens may have greater utility than other markers of immune development (e.g., cytokines or T cell populations).
- All panelists agreed the data suggest OPN has no impact on BW growth in early life.
- All panelists agreed that the data on gastrointestinal development did not suggest adverse impacts on development, though an understanding of the mechanisms of some changes is nascent.
- All panelists agreed that despite potential crossing of the BBB, none of the behavioral or physiological outcomes suggested that dietary bmOPN has an adverse effect on neurodevelopment. Some panelists suggested no further research is needed to characterize the safety of bmOPN in this respect. Others suggested that further measures of cognitive and behavioral development would be the most clinically relevant outcomes to assess the effects of dietary bmOPN on brain development.
- The panel concluded that functional bioequivalence appeared high given the similarity in results comparing bmOPN, recombinant bovine/human, and murine OPN on response to LPS and behavioral performance.

## Clinical evidence

One clinical trial has investigated the effects of orally ingested bmOPN and immune development in infants (79). In this double-blind randomized trial, 279 infants were BF or fed a standard formula with 15 (F0), 65 (F65), or 130 (F130) mg/L bmOPN (Lacprodan OPN-10) between 1 and 6 months of age (analyzed to contain 14, 72, and 150 mg OPN/L formula, respectively). Some infants in the F65 and F130 group started consumption of the experimental formulas prior to 1 month of age (personal communication with authors). Only small amounts of complementary foods were recommended to be introduced between 4 and 6 months of age. Of the clinically relevant endpoints identified by Callahan et al. (69), Lönnerdal et al. noted no differences between formula-fed groups in anthropometry/growth and adverse effects (79). At 6 months, the IgG response to tetanus (infants were vaccinated against diphtheria, pertussis, and tetanus at 4 months of age) was equivalent between F130, F0, and BF groups, with fewer antibodies in the F65 than the F0 group (79). Breastfed infants had fewer episodes of pyrexia than FF infants. Among the FF infants, the F0 group had a significantly higher incidence and prevalence of pyrexia than the BF infants, whereas there was no significant difference between the F65 or F130 groups and the BF infants. These data are consistent with epidemiological findings that the concentration of mother’s hmOPN is inversely associated with the number of infant hospital admissions due to fever in the first 3 months (*N* = 85) (36). Ultimately, the clinically relevant immune endpoints indicated no or minimal differences between the F0 and F135 groups (79).

Of the immune markers (cytokines and immune cell populations) at 6 months of age, Lönnerdal et al. reported no difference in plasma levels of IL-6, IL-8, IL-12, IL-15, and transforming growth factor β2 between the F130 and F0 groups. By repeated measures analysis over all timepoints, the F65 and F130 groups were lower in concentrations of TNF-α (though not at 6 mo) and IL-10, and higher in IL-2, than in the F0 group (79). To better understand if such changes in cytokines corresponded with changes in T cell populations, West et al. performed a secondary analysis of the same subjects investigating peripheral blood immune cells via flow cytometry (80). West et al. report that the F130 and F0 groups appeared statistically equivalent for all outcomes measured except for greater T cells on average (averaged over all timepoints) in the F130 group (though not different from the BF group), in peripheral blood mononuclear cells (81). Although not statistically compared within each timepoint, the F130 group had higher T cells at 1 month of age than F0 and F165 groups, which decreased over 1 to 6 months and appeared more similar to all other groups by 6 months of age than at 1 month. This pattern echoed the concept of physiological “convergence” by 12 months of age between formula-fed and BF infants described by Callahan et al. (69). Greater T cell populations (and cytokines) in the F130 group at one month of age may have reflected consumption of formula by some infants prior to sampling. Otherwise, the F130 group did not differ from F0 in: concentration of circulating white blood cells, lymphocytes, monocytes, eosinophils, basophils, or neutrophils; immune cell composition of T helper or T cytotoxic cells; or proportion of naïve T cells (CD45T0+CD3+), memory/activated T cells (CD45R0+), HLA-DR T cells (HLA-DR+CD3+), double-positive T cells (CD3+CD4+CD8+), αβ T cells, γδ, T cells, B cells, or natural killer cells (81). Ultimately, this clinical trial demonstrated high similarity in clinically relevant immune endpoints between standard formula and OPN supplemented formula, with OPN-fed infants displaying modestly lower (but statistically insignificant) incidence of fever than those fed a standard formula. Although some markers of immune development (i.e., IL-2, IL-10, and T cell populations) differed, the overall phenotypes shifted toward that of the BF group by 6 months of age.

**Panel conclusions** Although the trial did not extend beyond 6 months, given the similarity in clinical endpoints, the panel concluded that OPN-supplemented infants did not appear to have a different developmental trajectory than those fed standard formula.

## Osteopontin and the infant formula matrix

The interactions between bioactive constituents within the infant formula matrix have been identified by the FDA as an important gap to address when assessing safety (17). For OPN, available preclinical research on this topic has focused primarily on the interaction between OPN and LF. Both OPN and LF are whey proteins present at up to 10-times higher in concentration in human milk compared to bovine milk (82), and LF is 10-times higher in milk than OPN (83). As oppositely charged ions, it has been hypothesized that OPN and LF may have a carrier-like relationship. Supporting this hypothesis, Yamniuk et al. demonstrated that LF and OPN form a complex in milk at a ratio of 3:1 (83). The LF-OPN complex has demonstrated greater resistance to digestion (84, 85), binding and uptake by human intestinal cells (84), promotion of proliferation and differentiation of intestinal cells (84, 85), anti-bacterial activity (84, 85), and stimulation of IL-18 compared to LF or OPN alone (84, 85). The LF-OPN complex consisting of iron-free LF (apo-LF) and calcium-bound OPN (holo-OPN), which are the predominant forms of LF and OPN in human milk, is resistant to digestion and has the strongest effect on cell proliferation compared with other forms (e.g., holo-LF and apo-OPN) (86). The LF-OPN complex may act by binding to cell surface receptors to activate the PI3K/Akt signaling pathway (86). While further research may elucidate the modes of action of the LF-OPN complex, it is clear that the complex does not impair the function of the individual proteins, and that they act in combination. Less is known regarding OPN’s interaction with other components, though the combination of OPN, 2′-fucosyllactose, and docosahexaenoic acid was more effective at promoting maturation and differentiation of oligodendrocyte progenitor cells *in vitro* than either of the 3 individually (87).

## Conclusion

The panel concluded that the existing data establish the following points: exposure to hmOPN is quantifiable through 6 months of age; dietary bmOPN comprises <5% of total circulating OPN and is cleared from plasma within 24 h; preclinical studies demonstrate no effect of dietary bmOPN on growth; dietary bmOPN does not appear to alter the trajectory of immune development; neither the gastrointestinal nor brain-related data demonstrate an adverse impact of bmOPN consumption; and multiple forms of OPN demonstrate high functional bioequivalence. For future research, the panel recommended prioritization of trials measuring a comprehensive set of clinically relevant outcomes on immunity and cognition to confirm the safety of bmOPN over that of further research on ADME. Such research would clarify the reproducibility of current findings, increase the confidence of these conclusions, and contribute to the body of evidence on safety.

## Author contributions

SF: Conceptualization, Data curation, Formal analysis, Project administration, Supervision, Visualization, Writing – original draft, Writing – review & editing. SR: Writing – original draft, Writing – review & editing, Validation. SD: Writing – review & editing. OH: Writing – review & editing. RJ: Writing – review & editing. BL: Writing – review & editing, Conceptualization, Supervision. JN: Writing – review & editing. LS: Writing – review & editing. ES: Writing – review & editing. CW: Writing – review & editing. RK: Writing – review & editing. JW: Writing – review & editing, Conceptualization, Supervision.
